# Primary Thyroid NUT Carcinoma With High PD-L1 Expression and Novel Massive *IGKV* Gene Fusions: A Case Report With Treatment Implications and Literature Review

**DOI:** 10.3389/fonc.2021.778296

**Published:** 2022-01-19

**Authors:** Juan Zhou, Miao Duan, Qiong Jiao, Chunyan Chen, Aiyan Xing, Peng Su, Juan Tang, Hui Zhang, Zhiyan Liu

**Affiliations:** ^1^ Department of Pathology, Shanghai Jiao Tong University Affiliated Sixth People’s Hospital, Shanghai, China; ^2^ Department of Pathology, School of Basic Medical Sciences, Cheeloo College of Medicine, Shandong University, Jinan, China; ^3^ Department of Pathology, Qilu Hospital of Shandong University, Jinan, China

**Keywords:** NUT carcinoma, thyroid, cytopathology, *IGKV* gene fusions, *BRD4-NUT* fusion, PD-L1

## Abstract

**Background:**

Nuclear protein in testis (NUT) carcinoma (NC) is a rare and aggressive undifferentiated carcinoma that typically arises from midline supradiaphragmatic structures. It is uniquely driven by a *NUT* gene rearrangement on chromosome 15q14. Few thyroid NCs have been reported and there are no established treatment guidelines for NUT carcinoma.

**Method:**

Ultrasound-guided fine needle aspiration smear was performed for the preoperative diagnosis of thyroid lesions. Cytopathology, histology, and immunochemical staining all indicated NC. Fluorescence *in situ* hybridization (FISH), qRT-PCR, and next-generation sequencing (NGS) were used to analyze the genetic characteristics of NC.

**Results:**

We describe a rare case of thyrogenic NC in a 38-year-old male with cytological, histological, immunohistochemical, and genetic features. Cytological smears and histopathological specimens showed typical features of NC. Immunohistochemistry confirmed strong immunoreactivity with NUT, EMA, P63, TTF-1, and c-myc. CK19 was positive exclusively in sudden keratosis. No immunoreactivity was found for neuroendocrine markers. FISH was applied to isolate the *NUT* gene on chromosome 15q14. The NGS results revealed a *BRD4-NUT* gene fusion, which was further confirmed by RT-qPCR. Structural variation (SV) of *NUTM1* occurred in the exon region, and the mutation site was 15q14. Moreover, *BRD4* single-nucleotide variation (SNV) occurs in the 3′ UTR at mutation site 19p13.12. The PD-L1 combined predictive score was over 30%. The patient received chemotherapy, followed by programmed cell death 1 (PD-1) inhibition with camrelizumab, and died 10 months after surgery.

**Conclusion:**

Thyroid NC is an extremely rare and fatal malignant tumor. It is necessary to consider NC when squamous differentiation is observed cytologically or histologically. NGS is an effective tool for obtaining the final diagnosis and obtaining a better understanding of tumor pathogenesis. A large number of *IGKV* gene fusions in addition to the *BRD4-NUT* fusion may play a role in the pathogenesis and immunotherapy response of NC. Immunotherapy for NC remains to be explored due to the rarity of this aggressive malignancy.

## Introduction

Nuclear protein in testis (NUT) carcinoma (NC) is an extremely aggressive carcinoma with a high fatality rate and a median overall survival of 6.7 to 9.5 months (1–5). NC mainly occurs in the head and neck (especially the sinus tract) and the thorax (especially the mediastinum). It is important to recognize NC for therapeutic and prognostic reasons (4, 6). Approximately 70% of NCs occur due to NUT gene rearrangement (also known as the *NUTM1* gene) on chromosome 15q14, which is attributed to *NUT* gene fusion with BRD4 on chr19p13.1, resulting in (15;19) (q14;p13.1) (7). Meanwhile, the fusion mode of the other 30% of NCs are *BRD3-NUT*, *NSD3-NUT*, *ZNF532-NUT*, or *ZNF592-NUT* (7). Ectopic histone hyperacetylation is induced by the association of NUT, BET protein, and p300, resulting from the NUT fusion, leading to different NC genotypes (8). These fusion genes promote gene inactivation, which leads to the dedifferentiation and rapid proliferation of squamous cells.

Here, we first report the cytological, pathological, and molecular features of thyroid NC and the response to combined chemotherapy and PD-1 inhibition in NUT carcinoma. To the best of our knowledge, this is a rare case of a thyroid NC patient who achieved a prolonged response from a combination treatment regimen consisting of chemotherapy and immunotherapy.

## Materials and Methods

### Cell Smear and Tissue Sections

Ultrasound-guided fine needle aspiration biopsy was applied as a preoperative diagnosis of thyroid nodules. Direct cell smears of inhaled specimens were fixed with 95% ethyl alcohol, and surgical samples were fixed in neutral formalin. Cell smears and paraffin-embedded tissue sections were stained with hematoxylin–eosin. These slides were reviewed by HZ and ZL.

### Immunostaining

Cell smear and paraffin section specimens were immunostained. Testicular tissue was used as a positive control for the NUT antibody, and skin tissue was used as a positive control for P63. Phosphate-buffered saline was used instead of the antibodies as a negative control. Detailed antibody data are summarized in **Table S1 of the Appendix**.

### Fluorescence *In Situ* Hybridization

Fluorescence *in situ* hybridization (FISH) was used to analyze the 5-μm tissue sections fixed with formalin and embedded with paraffin. Dual-color probes for the 15q14 breakpoint flanking *NUT* included the telomeric bacterial artificial chromosome clone *SHGC-110339* (211 bp, green) and the centromeric clone *RH54191* (306 bp, red). A fluorescent probe (F.01264, LBP, Guangzhou China) was hybridized with nucleic acids in the tissue sections to observe the precise location of nucleic acids in the sequence under a fluorescence microscope.

### Quantitative Real-Time PCR Detection System

DNA was extracted from cancer and non-cancer tissues using DNA extraction kits (8.0223501X036G, AmoyDx, Xiamen, China). Quantitative real-time PCR (qRT-PCR) was carried out in a 10-μl mixture of 5 μl of SYBR Green, 0.1 μl of F primer, 0.1 μl of R primer, 1 μg of cDNA, and nuclease-free water. The primer design was based on *BRD4-NUT* fusion information and consisted of the *BRD4-NUT*-F sequence (5′-AGTCATCCAGCACCACCATTC-3′) and *BRD4-NUT*-R sequence (5′-GGTCTGGTGGGTCAGAAGTT-3′). To compare the difference in gene content between normal and cancerous tissues, the following formula was applied: 2^−ΔΔCT^ is applied, where ΔΔCT = (CT^target^ − CT^control^) cancer − (CT^target^ − CT^control^) normal.

### Next-Generation Sequencing

Genomic DNA (thyroid tumor tissue and blood) and total RNA (thyroid tumor tissue) were isolated using an *AllPure* total DNA/RNA micro kit (Magen, China) according to the instructions of the manufacturer.

Whole-genome sequencing (WGS) libraries were generated using the *TruSeq* DNA LT Sample Prep Kit v2 (Illumina). *RNAseq* sequencing was performed on cDNA libraries prepared from PolyA+ RNA extracted using the Illumina *TruSeq* protocol for mRNA, and libraries were constructed using the Illumina 3000 platform (2 × 150 bp). The average coverage of tumor tissue and non-tumor tissue samples was 30×.

The raw WGS sequence reads were clipped using Skewer (v0.2.2), and reads were aligned with GRCh37 by BWA-MEM. We used Control-free C (v9.1) for copy number variation (CNV) detection and Manta (0.29.6) for structural variant (SV) calling.

RNAseq raw sequence reads were trimmed using *Skewer* (v0.2.2). *FastQC* (v0.11.2) was used for quality control of RNA sequencing data. Then, mapping to the GRCh38 was performed using STAR 2.5. Fusion genes were called with STAR-Fusion.

Fusion visualization was created from scripts using *Integrated Genome Viewer* version 2.1.30 (Broad Institute, Cambridge, MA, USA), which allows visualization of supported reads along the alignment of the composite reference fusion.

## Results

### Clinical Features

**Figure 1:**
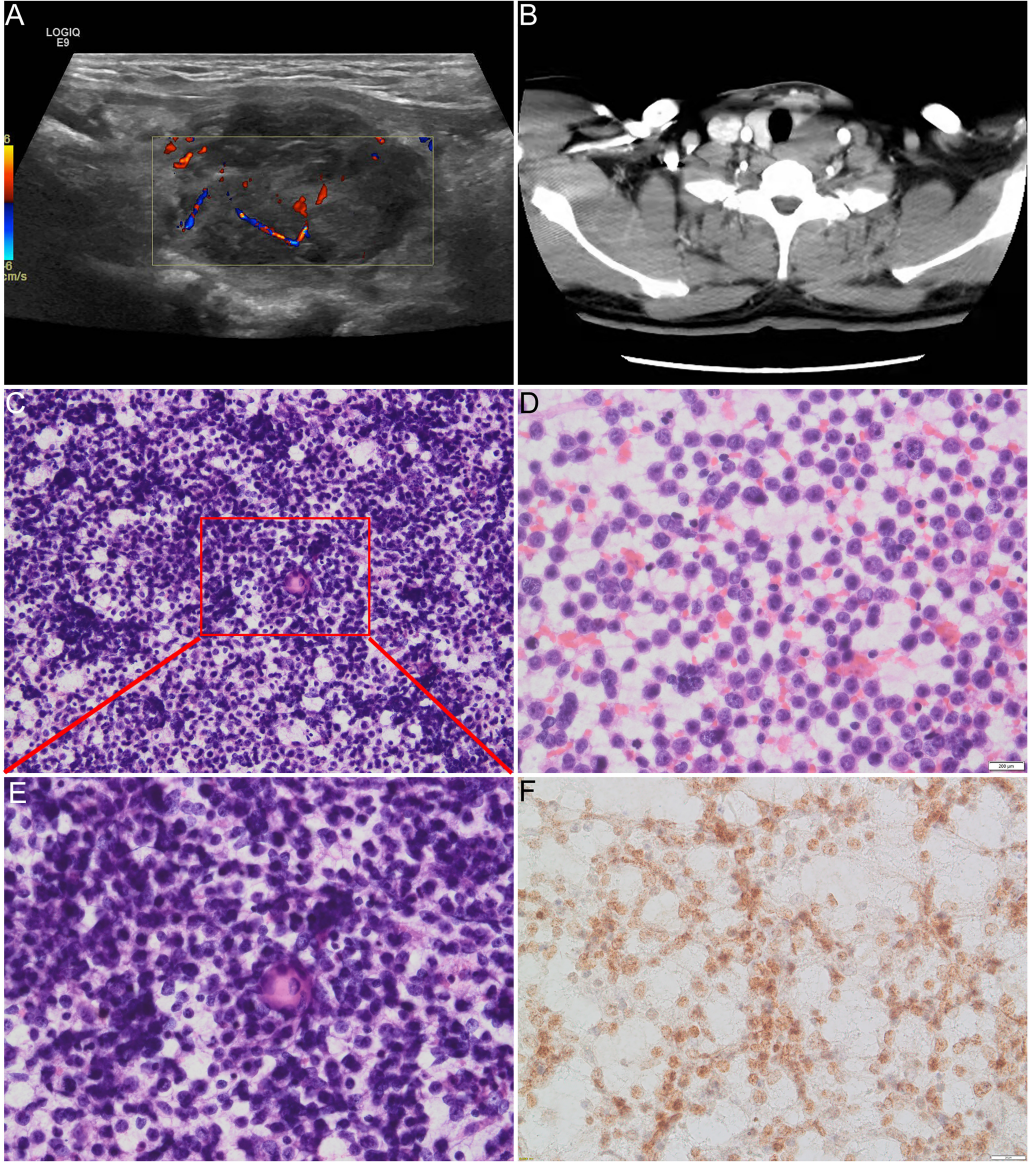
**(A)** Ultrasound examination revealed a heterogeneous nodule in the left lobe of the thyroid. **(B)** CT examination revealed 4-cm low-density lesions in the left lobe of the thyroid gland. **(C, D, E)** Direct cell smears show abundant cells with diffuse distribution of small- to medium-sized atypical monolayer protocells, and the foci of sudden keratosis could be seen from low magnification in **(C)** and high magnification in **(E)**. **(E)** Tumor cells have round to oval nuclei with bare and scanty cytoplasm and vague to prominent nucleoli. The chromatin of the nucleus varies from mostly light open chromatin to hyperchromatic. **(F)** Positive immunoreaction with NUT protein in the nucleus of the carcinoma cells.

A 38-year-old man was evaluated by a doctor at Qilu Hospital of Shandong University (Shandong, China) for a hard and painless neck nodule that had persisted for approximately 3 months. Ultrasound examination revealed a 3.8 × 2.7-cm irregular heterogeneous nodule in the left lobe of the thyroid gland with multiple point-like strong echoes (**Figure 1A**), which was further confirmed by CT (**Figure 1B**).

### Cytopathologic and Immunochemical Features

Direct cell smears revealed an abundance of diffuse, small- to medium-sized, atypical dispersed monolayers of primitive cells with a few lymphocytes in the background (**Figures 1C–E**). The neoplastic cells had round to elliptic nuclei with sparsely exposed cytoplasm and indistinct nucleoli. The chromatin of the nucleus varied from mostly light, open chromatin to hyperchromatin (**Figure 1E**). Mitosis was rare, and atypical mitosis was not seen. Sudden keratosis was observed (**Figures 1C, E** **)**. A positive immunoreaction with the NUT antibody was found in the nuclei of the carcinoma cells (**Figure 1F**).

**Figure 2:**
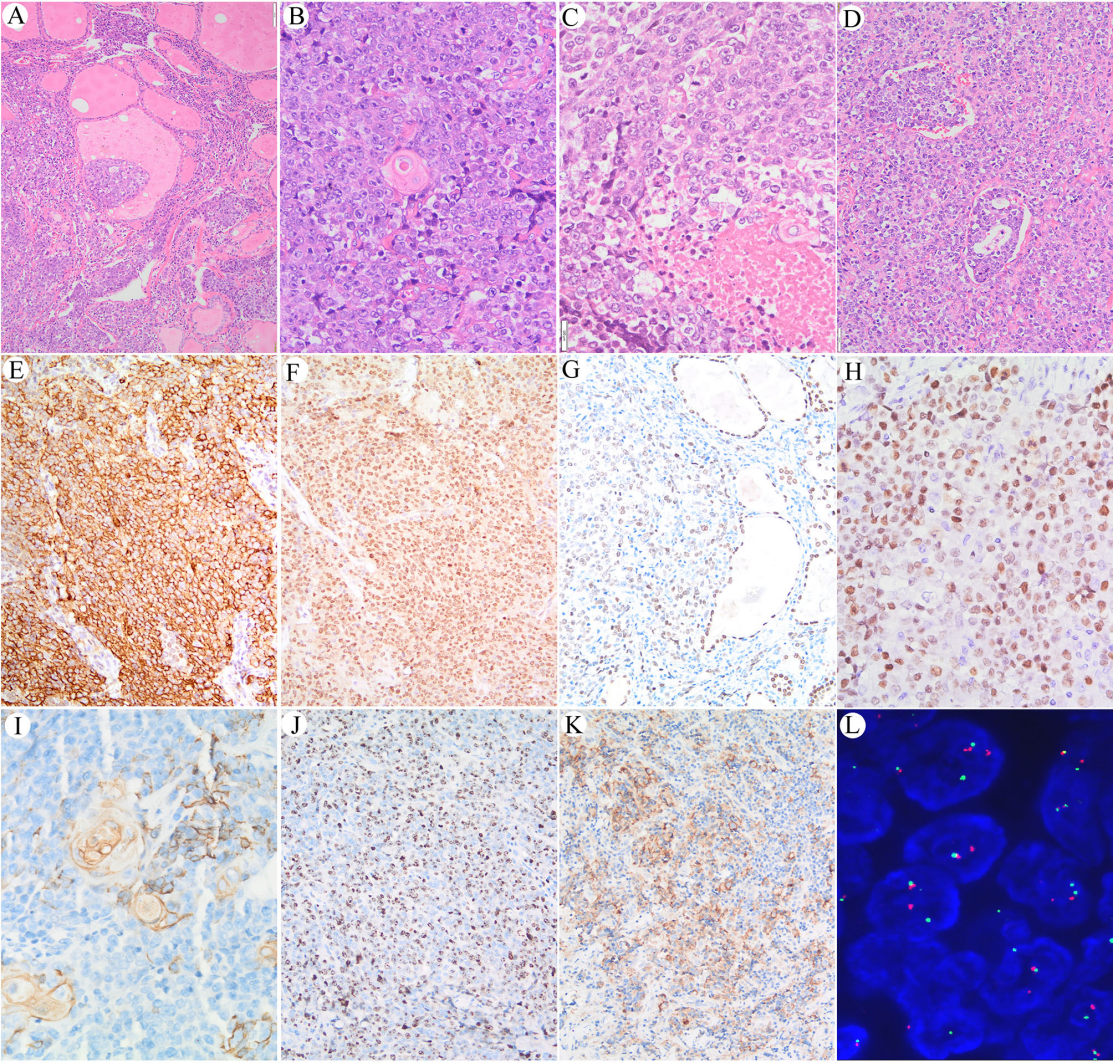
**(A)** Nests and sheets of primitive cells with high nuclear/cytoplasmic ratios infiltrate surrounding normal thyroid tissue. **(B)** Sudden keratosis foci could be seen. **(C)** Coagulative necrosis. **(D)** Lymphoid vessel involvement. **(E)** Immunoreaction with EMA in the cell membrane of the NUT carcinoma cells. **(F)** Immunoreaction with NUT protein in the nucleus of the NUT carcinoma cells. **(G)** Positive immunoreaction with TTF-1 in the NUT midline carcinoma cells; internal positive control of follicular thyroid epithelium is seen in the right part of the picture. **(H)** Positive immunoreaction with c-myc was found in approximately 80% of the carcinoma cells. **(I, J)** Immunoreaction with CK19 in sudden keratoses and a few NUT midline carcinoma cells. **(J)** The Ki-67 labeling index is high. **(K)** Positive immunoreaction with PD-L1 in carcinoma cells and a few inflammatory cells. **(L)** FISH shows splitting of the translocated *NUTM1*.

The corresponding surgical sections showed the typical appearance of NC. Nests and sheets of primitive cells with a high nuclear/cytoplasmic ratio infiltrate surrounding normal thyroid tissue were present (**Figure 2A**). There was squamous differentiation with prominent red medium-sized nuclei, suggestive of NC (**Figure 2B**). Coagulative necrosis and lymphatic vascular involvement were frequently observed, with mitotic rates as high as 3–6/5 high visual fields (**Figures 2C, D** **)**. The carcinoma cells were typically undifferentiated with a relatively uniform nucleus size, irregular nuclear membrane, and uneven chromatin.

Positive immunoreaction with EMA and the NUT protein was observed in the carcinoma cells but not in the sudden keratoses (**Figures 2E, F** **)**. A positive TTF-1 immune response was observed in approximately 60% of the carcinoma cells, suggesting that the tumor originated from follicular thyroid cells (**Figure 2G**), whereas only a few carcinoma cells were PAX-8 positive. A positive c-myc immunoreaction was found in approximately 80% of the carcinoma cells (**Figure 2H**). The CK19-positive immune response in the cytoplasm was mainly observed in cells with squamous differentiation (**Figure 2I**). The Ki-67 labeling index was approximately 60% (**Figure 2J**). The PD-L1 combined proportion score was 30% (**Figure 2K**). No immunoreactivity with P53, TG, CD30, CD5, or ALK was observed.

### Complex Chromosomal Rearrangements Involving *BRD-NUT* Oncogenes

Dual-color FISH was further applied to confirm the split-apart translocated *NUTM1*, as shown in **Figure 2L**. However, although split-apart signals were confirmed, the genes fused with *NUTM1* remained unknown, and WGS was performed.

**Figure 3:**
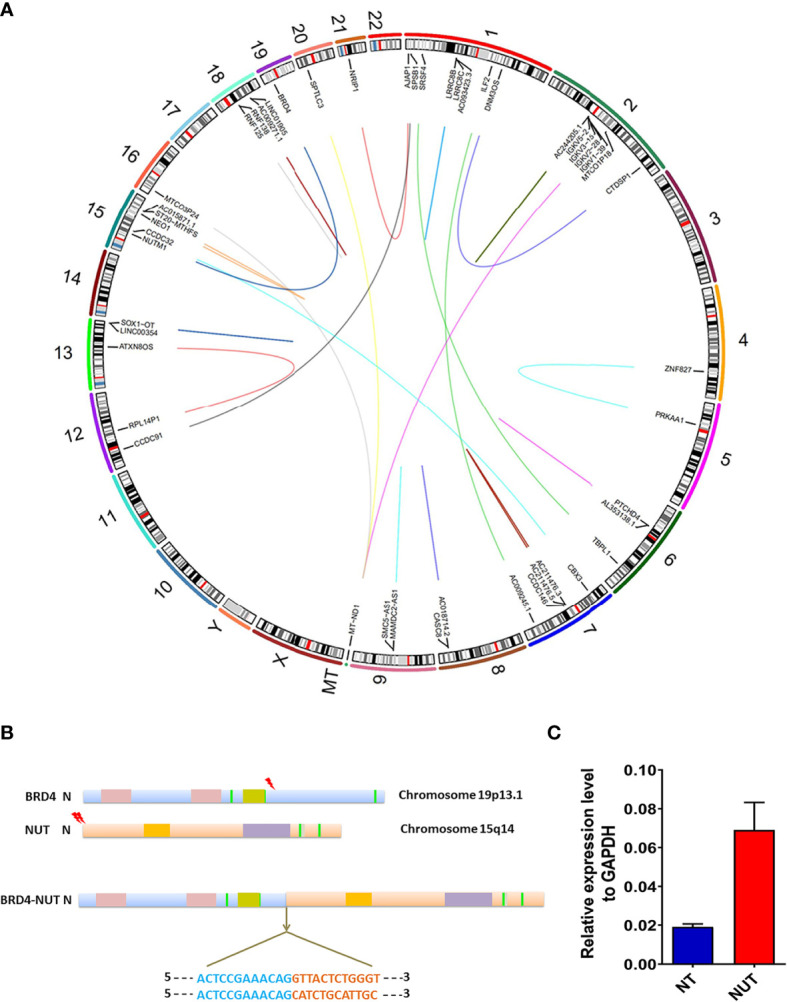
Genomic rearrangements of BRD-NUT gene fusion and detailed introduction. **(A)** Circos plot showing the genetic variants identified in a thyroid NUT carcinoma genome. The corresponding genes are labeled inside the circus. The arc connecting the chromosomes signifies translocation detected in sequencing data. **(B)** Schematic ideograms show gene fusions between *BRD4* (chr.19p13.1) and *NUTM1* (hr. 15q14). The arrowheads indicate the breakpoints of *BRD4* (exon 11) and *NUTM1* (exon 2/3). Each base in the fusion plot is drawn 5′ to 3′. **(C)** RT-qPCR showed that the expression level of *BRD4-NUT* fusion in cancer tissue was 3.58 higher than that in normal tissue, further confirming the results of WGS.

Large structural variations (SVs) and DNA copy number alterations were investigated using WGS. Based on *Manta* 0.29.6 bioinformatics analyses and visual inspection, 318 genes with genetic SVs were screened out, including 211 in exon segments, 75 in intergenic regions, and 25 in introns. Six SVs were located in the intron region of UTR3 and ncRNA, and the last SV was located in UTR5. Structural variation of *NUTM1* occurred in the exon region at 15q14, while *BRD4* SV occurred in the UTR3 region at 19p13.12 (**Figures 3A, B** **)**.

The WGS results revealed multiple gene fusions, as shown in **Table 1**. According to the fusion information analysis, the *BRD4-NUT* fusion gene is formed by the fusion of exons 2 and 3 of the *NUT* gene and exon 11 of the *BRD4* gene (**Figure 3A**). qRT-qPCR showed that the expression level of *BRD4-NUT* fusion in the cancer tissue was 3.58 higher than that in the normal tissue, further confirming the results of WGS (**Figure 3C**). Interestingly, further analysis revealed that there were two fusion modes of *BRD4-NUT* in the current patient. Although two NUT breakpoints were identified (chr15:34640170:+; chr15:34638143:+), there was a single *BRD4* breakpoint (chr19:15364963:−) (**Table 1**). The aforementioned breakpoints differ from previous findings that both the *NUT* (chr15:34629526) and *BRD4* (chr19:15359244) breakpoints occur at a single location (27). We first discovered a large number of *IGKV* gene fusions through RNA sequencing (**Table 1**), which mainly play a role in humoral immunity (9).

**Table 1:** Multiple gene fusions.

Fusion name	Left breakpoint	Right breakpoint
BRD4–NUTM1	chr19:15364963:−	chr15:34640170:+
BRD4–NUTM1	chr19:15364963:−	chr15:34638143:+
IGKV1-39–AC096579.13	chr2:89619384:−	chr2:89161074:−
IGKV1-39–AC096579.13	chr2:89619378:−	chr2:89161068:−
IGKV1-39–AC096579.13	chr2:89619382:−	chr2:89161433:−
IGKV2-28–AC096579.13	chr2:89521180:−	chr2:89161074:−
IGKV2D-28–AC096579.7	chr2:89999557:+	chr2:89160435:−
IGKV2D-28–AC096579.7	chr2:89999563:+	chr2:89160764:−
IGKV2D-28–AC096579.7	chr2:89999559:+	chr2:89160115:−
IGKV2D-28–AC096579.7	chr2:89999557:+	chr2:89161435:−
IGKV2D-28–AC096579.7	chr2:89999557:+	chr2:89161074:−
IGKV3-20–AC096579.13	chr2:89442061:−	chr2:89161074:−
IGKV3-20–AC096579.13	chr2:89442052:−	chr2:89161068:−
IGKV3-20–AC096579.7	chr2:89442058:−	chr2:89160117:−
IGKV3-20–AC096579.7	chr2:89442058:−	chr2:89161435:−
IGKV3-20–AC096579.7	chr2:89442543:−	chr2:89157196:−
IGKV3-20–AC096579.7	chr2:89442058:−	chr2:89160435:−
IGKV3-20–AC096579.7	chr2:89442055:−	chr2:89161074:−
IGKV3-20–AC096579.7	chr2:89442060:−	chr2:89160769:−
IGKV3-20–AC096579.13	chr2:89442055:−	chr2:89161074:−
IGKV3-20–AC096579.13	chr2:89442055:−	chr2:89161435:−
IGKV4-1–AC096579.7	chr2:89185671:+	chr2:89161432:−
IGKV4-1–AC096579.7	chr2:89185668:+	chr2:89161074:−
IGKV4-1–AC096579.7	chr2:89185670:+	chr2:89160433:−
IGKV4-1–AC096579.7	chr2:89185136:+	chr2:89157196:−
IGKV4-1–AC096579.7	chr2:89185668:+	chr2:89160117:−
IGKV5-2–AC096579.7	chr2:89197299:+	chr2:89161074:−
IGKV5-2–AC096579.13	chr2:89197299:+	chr2:89161074:−
TIMM23–LINC00843	chr10:51606988:−	chr10:51732772:+
TIMM23–PARGP1	chr10:51606988:−	chr10:51732772:+
RNF138–RNF125	chr18:29672849:+	chr18:29648261:+
RP4-769N13.6–GPRASP2	chrX:101860581:+	chrX:101968710:+
ARMCX5–GPRASP2	chrX:101860581:+	chrX:101968710:+
PDE6B–PPP2R2D	chr4:619882:+	chr10:133747960:+
AC004878.3–CCDC146	chr7:74949901:−	chr7:76866264:+
UPK3B–CCDC146	chr7:76648314:+	chr7:76866264:+
AC009245.3–ILF2	chr7:137406958:+	chr1:153634934:−
RP11-680G10.1–GSE1	chr16:85391249:+	chr16:85682158:+
CTD-2008L17.1–RP11-456O19.2	chr18:53560673:+	chr18:53717311:+
AJAP1–NRIP1	chr1:4772759:+	chr21:16415895:−
CCDC91–SPSB1	chr12:28515448:+	chr1:9427507:+
CTDSP1–DNM3OS	chr2:219267128:+	chr1:172113577:−
LRRC8B–LRRC8C	chr1:90050348:+	chr1:90152029:+
NEO1–ST20	chr15:73558752:+	chr15:80200026:−
NEO1–ST20-MTHFS	chr15:73558752:+	chr15:80200026:−
RP11-96H19.1–RP11-446N19.1	chr12:46781755:+	chr12:47046173:+
RP4-535B20.1–JAK1	chr1:65533287:−	chr1:65352024:−
SMC5-AS1–MAMDC2-AS1	chr9:72831202:−	chr9:72728776:−
SRSF4–TBPL1	chr1:29495015:−	chr6:134301220:+
ZNF827–PRKAA1	chr4:146791397:−	chr5:40777688:−
RP4-565E6.1–HYDIN	chr1:146126404:−	chr16:71196633:−
CTD-2008L17.1–RP11-456O19.2	chr18:53560673:+	chr18:53763306:+
AC004878.3–CCDC146	chr7:74953040:−	chr7:76871008:+

Although SNVs and CNVs were present in the NC genome, NC-associated promoters and suppressor genes were rarely affected, suggesting that they were primarily passenger events (**Supplementary Table 1**, **Data Sheet 2**).

### Treatment and Patient Outcome

**Figure 4:**
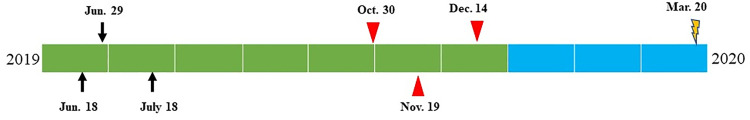
Time diagram from June 2019 to March 2020 of the patient: on June 18, 2019, thyroid nodule was identified by ultrasound and CT examination; on June 29, 2019, thyroid fine-needle aspiration was performed, and poorly differentiated carcinoma was suggested by cytology; on July 18, 2019, total thyroidectomy and lateral cervical lymph node dissection were performed. Thyroid NUT carcinoma was confirmed by morphology, immunohistochemistry, and genetic alterations. The combined proportion score of PD-L1 revealed to be 30%. On October 30, 2019, the patient received combined chemotherapy and PD-1 inhibition therapy (epirubicin 140 mg/dl, paclitaxel liposomes 240 mg/d2, and carelizumab 200 mg/dl). On November 19, 2019, CT examination revealed lateral cervical lymph node metastasis and bone metastasis on the 8th rib and L2 vertebra. Zoledronic for anti-bone metastasis was added to the previous treatment plan. On December 14, 2019, physical examination revealed a 5-cm mass on the right subscapular corner of the patient. Core needle biopsy revealed metastatic NUT carcinoma with focal necrosis (<10%), suggesting a poor response to the treatment. PET-CT showed multiple bone metastases. Due to the rapid progression, the chemotherapy regimen adjusted to cisplatin 50 mg/dl, 40 mg/d2,3+, and etoposide 0.1 dl–5. The patient died on March 20, 2020.

The PD-L1 combined predictive score was over 30%. Therefore, the patient received a combined treatment regimen consisting of epirubicin, paclitaxel liposomes, and an engineered anti-programmed death-ligand 1 (PD-1) antibody (camrelizumab). However, distant metastases were confirmed gradually, as shown in **Figure 4**. The patient refused further treatment due to economic difficulties. He developed systemic symptoms and died 10 months after surgery.

### Previous Studies of NUT Midline Carcinoma

Ninety-one cases of NC have been described in the PubMed database, consisting of 47 females and 44 males, with a male to female ratio close to 1:1 (2, 4, 5, 10–15). The age distribution ranges from 0.1 to 81.7 years, which is consistent with the current research status of all age groups, and the disease primarily affects adolescents. *BRD-NUT* gene fusion plays an important role in the pathogenesis of NC. Over 87% of fusion patterns were *BRD4-NUT*, usually comprising *NUT* exon 3 and BRD4 exon 11. Exon 2 of the *NUT* gene associated with exon 14 of the *BRD4* gene and exon 2 of the *NUT* gene associated with exon 11 of the *BRD4* gene have been reported (7, 16). There are various forms of fusion residues, including *BRD3-NUT*, *NSD3-NUT*, *ZNF532-NUT*, and *ZNF592-NUT* (7). The diagnosis of NC depends on morphological observation and positive NUT protein immunoreactivity. FISH, PCR, and whole genome sequencing are gradually being applied to clinical diagnostics.

## Discussion

Few thyroid NCs have been reported in the English or Chinese literature. Here, we report a rare case of thyroid NUT carcinoma and its cytopathologic, immunochemical, and somatic genetic features in a patient who received a combined treatment regimen consisting of chemotherapy and immunotherapy and achieved a prolonged response.

NC has a non-specific cytomorphology that is similar to that of other primitive small round cell tumors or basaloid neoplasms (17–22). The present case presented as a hypercellular smear with primitive cells and only small necrotic foci. No cytoplasmic vacuoles or giant or multinucleated tumor cells were observed. Interestingly, typical abrupt keratosis was observed, which has not been described in previous reports (17–22).

NC is often diffusely positive for NUT, EMA, P63, and c-myc, which was also confirmed in this case. We showed a positive NUT immunoreactivity in cell smears and surgical specimens. The immunoreactivity with TTF-1 suggests that the tumor probably originated in the thyroid (14, 22). Curiously, the samples were negative for PAX-8 expression despite this marker being considered a better marker of thyroid origin than TTF-1 (23). One previous case of NUT carcinoma developed after thyroidectomy of extraordinary thyroid sclerosing mucoepidermoid carcinoma with eosinophilia. However, the negative immunoreactivity of this tumor with TTF-1 suggested that it may be not of thyroid origin (24).

The differential diagnosis of NC includes a series of dedifferentiated neoplasia, such as squamous cell carcinoma, small cell carcinoma, ectopic thymic carcinoma, Ewing cell carcinoma, and a new entity, SMARCB1 (INI-1)-deficient sinonasal carcinoma (25–28). Immunohistochemistry and molecular testing are helpful for the differential diagnosis (25–28). In the context of relatively differentiated or undifferentiated tumor cells, sudden squamous differentiation without transitional morphology suggests the diagnosis of NC. NC with or without keratinization is most likely to be misdiagnosed as squamous cell carcinoma because these tumors co-express CK5/6 and P63. Interestingly, we found that CK19 expression was positive in squamous keratosis but negative in the surrounding carcinoma tissue, which could be used in the differential diagnosis. Primitive neuroectodermal tumors (PNETs) have no squamous differentiation, and positive immunoreactivity with CD99 and FLI1, but not NUT, supports the diagnosis of PNET (28). *EWSR1* gene translocation is helpful to distinguish PNET from NC (28). Poorly differentiated olfactory neuroblastoma may show marked nuclear abnormalities, sparse or absent interstitial nerve fibers, and pseudo-chrysanthemum pattern structures (29). Neuroendocrine markers are expressed in tumor cells, and S-100 immune responses in the supporting cells help to confirm the diagnosis.

Overall, the *BRD4-NUT* fusion was confirmed by FISH, NGS, and qRT-PCR. There were two fusion modes of *BRD4-NUT* in the current patient. Two NUT breakpoints were demonstrated (chr15:34640170:+; chr15:34638143:+), and a single BRD4 breakpoint was found (chr19:15364963:−). These breakpoints differ from those identified in previous studies, in which the single breakpoints were identified in both NUT (chr15:34629526) and BRD4 (chr19:15359244). Wild-type NUT is expressed only in the testes, and approximately 70% of NCs arise due to translocations between the 3′ end of the *NUTM1* gene on chromosome 15q14 and the 3′ end of BRD4 on chr19p13.1 (7, 30). NUT interacts with p300 and activates the histone acetyltransferase activity of p300 (8). *BRD4-NUT* directly regulates two key genes, *MYC* and *TP63*. The fusion protein formed by transcription of the *BRD4-NUT* gene can resist cell differentiation and promote cell proliferation with the help of the BET protein (31). The strong immune response of c-myc and p63 is consistent with these hypotheses (31–33).

The median survival for NUT carcinoma patients ranges from 4.7 to 6.7 months, and over 80% of NUT carcinoma patients die within 1 year (1, 2). *BRD4-NUT* fusions have been reported to be the most common rearrangements in NC, and small molecules targeting BRD4 have been developed (34, 35). An oral BET inhibitor targeting BRD2/3/4/T is currently being evaluated in clinical trials, and two NC patients responded rapidly to it (8, 30). Unfortunately, the patient in this case was unable to receive a BET inhibitor, and he received chemotherapy (epirubicin and paclitaxel liposome) combined with a PD-1 inhibitor (camrelizumab). The disease improved after the first treatment but not after the subsequent therapy, and distant metastases rapidly developed. Recently, a 34-year-old NUT carcinoma patient was reported to have a complete response to a combined treatment regimen of chemotherapy, concurrent external-beam radiation, and the PD-1 inhibitor with pembrolizumab (36). However, the current patient did not receive external radiation; he refused further treatment due to economic difficulties and died 10 months after the surgery. In addition to *BRD-NUT* gene fusions, a large number of *IGKV* gene fusions were also found in addition to *BRD-NUT* gene fusions, and these fusions mainly play a role in humoral immunity. Those two cases indicated that NUT carcinoma patient may benefit from PD-1 inhibition therapy. However, data for more patients are needed to support this hypothesis.

Some SNVs and CNVs were also identified by NGS; however, the importance of those alterations in NC is still unknown. Some reports suggest that SNVs may be related to the aggressiveness of malignant tumors. *ADGRB3* can cause cognitive impairment and ataxia (37). *HTRA2* promotes breast cancer cell growth and invasion and pancreatic cancer cell apoptosis (38, 39). Mutations in *MUC16* indicate poor lung cancer prognoses and contribute to the progression of cervical cancer (40). *ITIH* is significantly reduced in breast, colon, and lung cancers. Overexpression of *CASS4* promotes invasion of non-small-cell lung cancer (40). Both *HLA-A* and *HLA-C* are involved in immune responses (41, 42). *ZNF229* may play a regulatory role in transcription. However, the relationship between the above genes and NUT carcinoma has not been reported.

In addition, several CNVs were observed in our case. The *WASH* complex acts as a regulator of LDL and HDL metabolism (43). *CACNA1B* mutations have been reported to be a distinct myoclonus-dystonia syndrome (44). *MUC6* has been reported in Alzheimer’s disease (45). The *KCNJ18* gene is associated with recurrent thyrotoxic periodic paralysis, and *RIMBP3* is involved in spermatogenesis. Whether these SNVs and CNVs play a role in the pathogenesis of NC needs to be investigated in more patients, but this is difficult to achieve due to the rarity of the disease.

In summary, NC is an extremely aggressive carcinoma that can originate in the thyroid. It is necessary to consider NC when poor differentiation is observed cytologically and histologically. NUT immunoreactivity helps confirm the diagnosis. NGS helps to better understand the pathogenesis of tumors. Surgery and radiotherapy are still the main treatment options for NC, and targeted therapy and immunotherapy remain to be explored.

## Ethics Statement

The studies involving human participants were reviewed and approved by the Ethics Committee of Qilu Hospital of Shandong University. The patients/participants provided their written informed consent to participate in this study.

## Author Contributions

HZ and ZL: conception and design of the work; acquisition, analysis, and interpretation of data; and revision of the manuscript critically for important intellectual content and scientific integrity. JZ and DM: drafting of the manuscript. QJ, CC, HX, PS, and JT: acquisition, analysis, and interpretation of data; and reading and revision of the manuscript critically for important intellectual content and scientific integrity. All authors contributed to the article and approved the submitted version.

## Funding

This work has been supported by the National Nature Science Foundation of China (Grant No. 81972500, and 81802914) and the Natural Science Foundation of Shandong Province, China (Grant No. ZR2019MH024), Grant from Innovation Program of STCSM (Shanghai Science and technology committee) (20Z11900304), and by research fund from Shanghai Jiao Tong University Affiliated Sixth People’s Hospital.

## Conflict of Interest

The authors declare that the research was conducted in the absence of any commercial or financial relationships that could be construed as a potential conflict of interest.

## Publisher’s Note

All claims expressed in this article are solely those of the authors and do not necessarily represent those of their affiliated organizations, or those of the publisher, the editors and the reviewers. Any product that may be evaluated in this article, or claim that may be made by its manufacturer, is not guaranteed or endorsed by the publisher.
