# Supplementary material for: Primary Thyroid NUT Carcinoma With High PD-L1 Expression and Novel Massive IGKV Gene Fusions: A Case Report With Treatment Implications and Literature Review
Source: Front Oncol. 2022 Jan 19;11:778296. doi: 10.3389/fonc.2021.778296 (PMC8807656; doi:10.3389/fonc.2021.778296)
Supplement: Supplementary file 2 [file DataSheet_2.docx]

Please download the raw figures below:

https://www.jianguoyun.com/p/Dei20_0QyO3tCRiVuI4E
